# Human leucocyte antigen class I in hormone receptor-positive, HER2-negative breast cancer: association with response and survival after neoadjuvant chemotherapy

**DOI:** 10.1186/s13058-019-1231-z

**Published:** 2019-12-11

**Authors:** Bruno Valentin Sinn, Karsten E. Weber, Wolfgang Daniel Schmitt, Peter A. Fasching, William Fraser Symmans, Jens-Uwe Blohmer, Thomas Karn, Eliane Tabea Taube, Frederick Klauschen, Frederik Marmé, Christian Schem, Elmar Stickeler, Beyhan Ataseven, Jens Huober, Gunter von Minckwitz, Barbara Seliger, Carsten Denkert, Sibylle Loibl

**Affiliations:** 1 Department of Pathology, Charité – Universitätsmedizin Berlin, corporate member of Freie Universität Berlin, Humboldt - Universität zu Berlin, and Berlin Institute of Health, Berlin, Germany; 2 Berlin Institute of Health (BIH), Berlin, Germany; 3 German Breast Group Forschungs GmbH, Neu-Isenburg, Germany; 4 Department of Gynecology, University Hospital Erlangen, Erlangen, Germany; 5 Department of Translational Molecular Pathology, The University of Texas – MD Anderson Cancer Center, Houston, TX USA; 6 Department of Gynecology with Breast Cancer, Charité – Universitätsmedizin Berlin, corporate member of Freie Universität Berlin, Humboldt - Universität zu Berlin, and Berlin Institute of Health, Berlin, Germany; 7 Department of Gynecology and Obstetrics, University Hospital Frankfurt, Frankfurt, Germany; 8 German Cancer Consortium (DKTK) Partner Site Berlin, Berlin, Germany; 9 Department of Gynecology and Obstetrics, University Hospital Heidelberg, Heidelberg, Germany; 10 Department of Gynecology and Obstetrics, University Hospital Schleswig-Holstein, Kiel, Germany; 11 Mammazentrum Hamburg, Hamburg, Germany; 12 Department of Gynecology and Obstetrics, RWTH Aachen, Aachen, Germany; 13 Department of Gynecology and Gynecologic Oncology, Kliniken Essen Mitte, Essen, Germany; 14 Department of Obstetrics and Gynecology, University Hospital, LMU Munich, Munich, Germany; 15 Department of Gynecology and Breast Medical Oncology, Universitätsklinikum Ulm, Ulm, Germany; 16 Institute of Medical Immunology, Martin Luther University Halle-Wittenberg, Halle, Germany; 17 Department of Pathology, University Hospital Marburg, Marburg, Germany

**Keywords:** Breast cancer, HLA, Tumor-infiltrating lymphocytes

## Abstract

**Background:**

Clinical application of cancer immunotherapy requires a better understanding of tumor immunogenicity and the tumor microenvironment. HLA class I molecules present antigens to CD8^+^ cytotoxic cells. Their loss or downregulation is frequently found in tumors resulting in reduced T cell responses and worse prognosis.

**Methods:**

We evaluated HLA class I heavy chain expression by immunohistochemistry in 863 biopsies (GeparTrio trial). Patients received neoadjuvant chemotherapy and adjuvant endocrine treatment if tumors were hormone receptor-positive (HR+). In parallel, the expression of HLA-A was analyzed using a microarray cohort of 320 breast cancer patients from the MD Anderson Cancer Center. We evaluated its association with clinical outcome, tumor-infiltrating lymphocytes (TILs), and immune cell metagenes.

**Results:**

In HR+/HER2− breast cancer, HLA class I heavy chain expression was associated with increased TILs and better response to chemotherapy (7% vs. 14% pCR rate, *P* = 0.029), but worse disease-free survival (hazard ratio (HR) 1.6 (1.1–2.4); *P* = 0.024). The effect was significant in a multivariate model adjusted for clinical and pathological variables (HR 1.7 (1.1–2.6); *P* = 0.016) and was confirmed by analysis of HLA-A in a microarray cohort. HLA-A was correlated to most immune cell metagenes. There was no association with response or survival in triple-negative or HER2+ disease.

**Conclusions:**

The study confirms the negative prognostic role of lymphocytes in HR+ breast cancer and points at a complex interaction between chemotherapy, endocrine treatment, and tumor immunogenicity. The results point at a subtype-specific and potentially treatment-specific role of tumor-immunological processes in breast cancer with different implications in triple-negative and hormone receptor-positive disease.

## Background

Interactions between cancer cells and the host immune system are important for development, evolution, and progression of cancer [1]. They influence response to therapy and survival of patients and modulating these effects offer new approaches for cancer therapy.

Immune checkpoint inhibitor (CPI) therapy can enhance therapy response in advanced triple-negative breast cancer (TNBC) [2], and several clinical trials are ongoing. For a successful implementation of such strategies in clinical practice, a better understanding of tumor-immunological effects is necessary.

Evidence suggests a role of the immune system in breast cancer, as the quantity of tumor-infiltrating lymphocytes (TILs) is associated with better response to neoadjuvant chemotherapy and better patient outcome [3, 4]. In a recent meta-analysis, TILs were associated with a higher probability of pathologic complete response (pCR) [5]. Abundant TILs were associated with a longer disease-free survival (DFS) in HER2+ breast cancer and TNBC. In contrast, TILs were associated with a shorter OS in patients with hormone receptor-positive, HER2-negative (HR+/HER2−) disease, pointing at differences according to breast cancer subtype.

Human leucocyte antigen (HLA) class I molecules are expressed on the surface of all nucleated cells and are encoded by the human leukocyte antigens HLA-A, HLA-B, and HLA-C. Their function is to present antigens to CD8+ cytotoxic T lymphocytes to recognize and eliminate infected or tumor cells [6]. Downregulation or loss of HLA class I expression is a frequent event in pathogen-infected and tumor cells as an effective mechanism to evade immune recognition [7, 8]. Deficient HLA class I expression can be mediated by promoter methylation [9], mutations in the HLA class I heavy chains (HC), b_2_-microglobulin (b_2_-m) or APM components, loss of heterozygosity of HLA gene loci, and transcriptional regulation [10, 11]. Molecular data suggest an impaired expression of components of the antigen presentation machinery (APM) as a mechanism of resistance to T cell response [12] and HLA class I abnormalities have been identified in tumors resistant to CPIs or adoptive T cell therapy [13].

Aim of this study was to evaluate the potential of HLA class I HC expression for prediction of response to neoadjuvant chemotherapy. To this end, we evaluated expression of HLA class I HC in a large cohort of breast cancer patients treated with anthracycline/taxane-based neoadjuvant chemotherapy (within the GeparTrio trial [14, 15]). We used a clinically annotated microarray cohort from the MD Anderson Cancer Center to validate the findings and investigate correlations with predefined metagenes [16] of different immune cell populations.

## Methods

### GeparTrio trial cohort

The neoadjuvant GeparTrio pilot [17] (NCT00544765) and main (NCT00544765) trials [14, 15] were prospective, randomized phase II and III trials including 2357 patients with breast cancer (cT2–4 cN0–3 cM0) recruited between 2001 and 2005. Patients received two cycles of docetaxel, adriamycin, and cyclophosphamide (TAC) and response was evaluated by ultrasound. Responders received four more cycles of TAC (pilot study) or were randomly assigned to four or six cycles of TAC (main study). Non-responders were randomized to receive either four cycles of TAC or four cycles of vinorelbine and capecitabine. HR+ was defined as ≥ 10% of tumor cells with estrogen receptor and/or progesterone receptor expression, as defined in the study protocol. HER2 positivity was determined by immunohistochemistry (HER2 score 3+) and in situ hybridization where appropriate (HER2/CEP17 ratio > 2.2). Endocrine treatment for 5 years was planned for patients with HR+ disease but was not part of the protocol. HER2 therapy was not available at that time. We used all samples with available material in the central biobank. Additional file 3: Table S1 lists the pathological and clinical characteristics of the patients. The extend of tumor-infiltrating lymphocytes (TILs) was estimated on H&E-stained whole tissue slides as the area of tumor cells (for intratumoral TILs) or the stromal area (for stromal TILs) that is covered by lymphocytes [3].

### Immunohistochemistry of HLA class I antigens

Immunohistochemical staining was performed on tissue microarrays using the anti-HLA class I antibody EMR-8-5 (dilution 1:600) recognizing the HLA class I HC HLA-A, HLA-B, and HLA-C (MBL, Woburn, MA, USA). The percentage of cells with membranous staining relative to all cancer cells was assigned to five categories: (0) 0%, (1) 1–9%, (2) 10–50%, (3) 51–80%, and (4) 81–100%. The intensity of staining was assigned into four categories: (0) negative, (1) weak, (2) moderate, (3) strong. The groups for percentage and staining intensity were multiplied resulting in the immunoreactive score (IRS) ranging from 0 to 12. Based on the distribution of staining levels and patient outcome, a cut point (IRS > 2) was selected to optimize separation of curves in Kaplan-Meier analysis. In addition, the percentage of positively stained tumor cells was used continuously to evaluate the effect of HLA immunohistochemistry on clinical endpoints without a defined cut-off.

### MD Anderson cancer center (MDACC) microarray cohort

Gene expression data and clinical information of the MD Anderson Cancer Center cohort [18] (Affymetrix U133A microarrays) was downloaded from the GEO repository (GSE25066). A total of 320 tumors were HR+/HER2− and patients received neoadjuvant chemotherapy and adjuvant endocrine treatment if HR+. HR positivity was defined as any number of stained cells. We applied an additional filter based on gene expression of ESR1 (probeset 205225_at) based on its bimodal distribution (> 10.45). In total, 267 patients met these criteria. Additional file 3: Table S2 lists the patient characteristics. HLA-A, HLA-B, and HLA-C metagenes were calculated as mean expression of the respective probesets (HLA-A: 215313_x_at, 213932_x_at; HLA-B: 211911_x_at, 209140_x_at, 208729_x_at, HLA-C: 208812_x_at, 216526_x_at, 214459_x_at, 211799_x_at). Unsupervised cut-offs were chosen by assigning the same fraction of cases to the groups with high (60%) and low (40%) expression as in the GeparTrio dataset (HLA-A > 14.24, HLA-B > 13.63, and HLA-C > 13.69). Probesets 205225_at (estrogen receptor 1) and 208079_s_at (Aurora kinase A) were used to evaluate their association with HLA-A. Immune cell metagenes were calculated as the mean expression of cell-type-specific genes [16].

### Statistical methods

Pathologic complete response (pCR) was defined as the absence of invasive cancer in breast and lymph nodes (ypT0/ypTis ypN0). In the MD Anderson cohort, response to chemotherapy was recorded using the residual cancer burden index [19]**.** Disease-free survival (DFS) was defined as time from study entry to local or distant recurrence or death from any cause, distant recurrence-free survival (DRFS) as the interval between diagnosis and distant recurrence or death from any cause, and overall survival (OS) as the time from study entry to death from any cause. To evaluate associations between clinical and pathological variables with HLA I, *χ*^2^ tests were used. Kaplan-Meier estimators and log-rank tests were used for survival analyses (R package *survival*). The independent prognostic value was assessed in multivariate Cox regression analyses and likelihood ratio tests. Statistical computations were performed in R version 3.3.1 (R Development Core Team, Vienna, Austria). Significance was based on *P* <  0.05 and 95% CI estimates.

## Results

### HLA class I expression in breast cancer

**Fig. 1:**
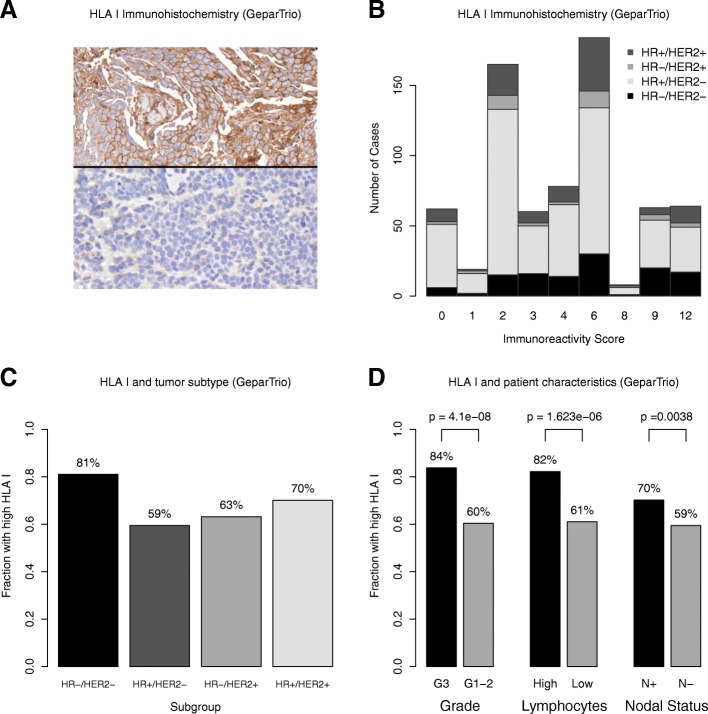
HLA class I HC immunohistochemistry in the GeparTrio cohort: HLA class I HC immunohistochemistry showed a membranous staining pattern. Examples of a positive case and a negative case are shown in the upper and lower half, respectively (**a**). The data distribution of the immunoreactive score (IRS) as a function of staining quantity (percentage) and quality (intensity) is shown. The colored bars represent the different breast cancer subtypes (**b**). HLA I was more frequently high in HR−/HER2− breast cancer as compared to other subtypes (**c**). It was also associated with higher tumor grade, tumor-infiltrating lymphocytes, and nodal status (**d**)

A total of 732 cases from the GeparTrio trial were analyzed for HLA class I HC expression. In total, 669 cases (91%) showed a positive staining; 63 were negative (9%, Fig. 1). We defined a cut-off to dichotomize HLA class I expression based on data distribution and patient outcome. This resulted in 480 cases with high expression (66%) and 252 (34%) with low expression across breast cancer subtypes. Additional file 3: Table S1 lists the patient and tumor characteristics of the GeparTrio study cohort that was evaluated for HLA class I HC expression (and Additional file 3: Table S2 the characteristics of the HR+/HER2− cohort).

### Association with clinical and pathological tumor characteristics

**Fig. 2:**
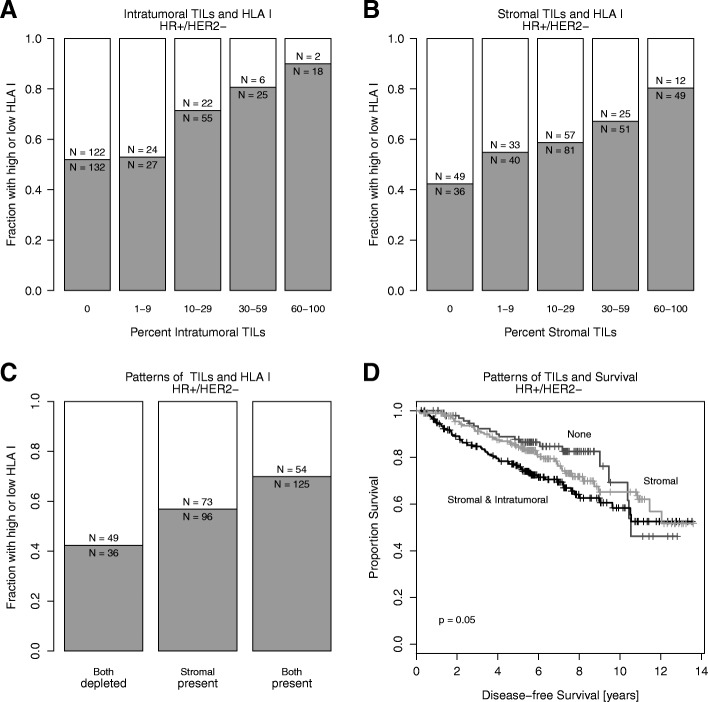
HLA class I HC expression in HR+/HER2− breast cancer and patterns of tumor-infiltrating lymphocytes (TILs): the fraction of tumors with high (gray) and low (white) HLA class I HC expression are shown according to the quantity of intratumoral TILs (**a**), stromal TILs (**b**) and the combination of the two patterns (**c**). TILs and their distribution and their association with progression-free survival in HR+/HER2- breast cancer (**d**)

The frequency of HLA class I HC expression was higher in TNBC compared to other subtypes (81% in TNBC, Fig. 1), in patients with node-positive disease (*P* = 0.004) and in tumors with a higher histological grade (*P* <  0.001). High levels of HLA class I HC expression was associated with extensive stromal TILs in the entire cohort (*P* < 0.001, Fig. 1) and within HR+/HER2− tumors (*P* < 0.001), where both the extent of intratumoral and stromal TILs were associated with high HLA class I HC expression (Fig. 2). There was no significant association with HER2 status, clinical T stage (T1–2 vs. T3–4), and histological subtype (no special type vs. lobular; data not shown).

### Association of HLA class I HC expression with clinical endpoints

**Fig. 3:**
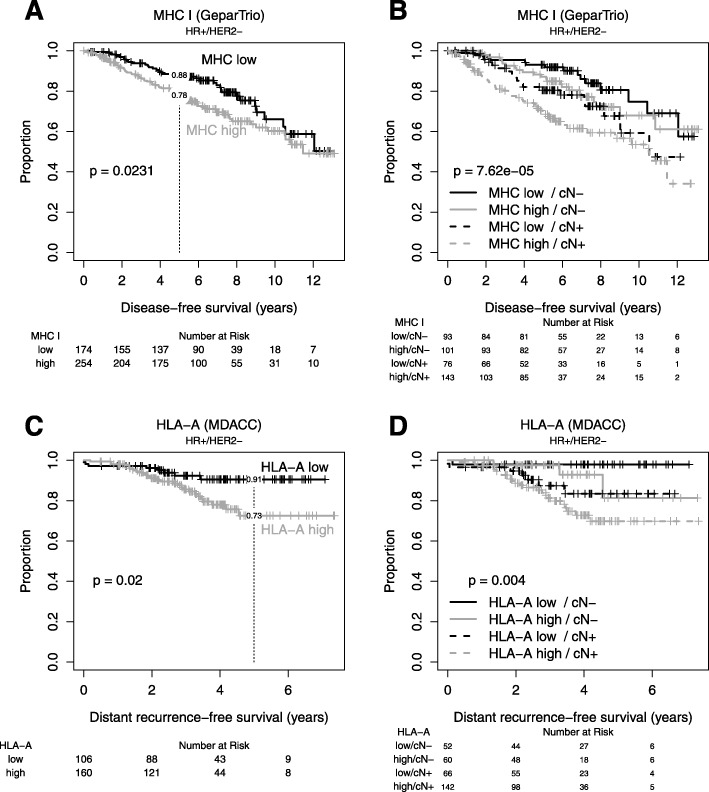
HLA class I HC immunohistochemistry in GeparTrio and HLA-A mRNA analysis in the MDACC cohort: in GeparTrio, HLA class I immunohistochemistry was associated with shorter disease-free survival (**a**, **b**) in HR+/HER2− tumors. For the HR+/HER2− microarray data, we chose a cut-off for HLA-A without prior knowledge of patients’ outcome to assign the same fraction of patients to each group as in the immunohistochemistry dataset. The effect on survival could be confirmed (**c**, **d**)

High HLA class I HC expression was associated with a higher rate of pathologic complete response (pCR) in patients with HR+/HER2− disease (7% vs. 14%, *P* = 0.029, Table 1), even after adjustment for clinical and pathological characteristics, but not in other subtypes (Table 1). High HLA class I expression was associated with shorter DFS in HR+/HER2− breast cancer (*P* = 0.024, Fig. 3, Table 1), but not in other subtypes (Table 1). In patients with HR+/HER2− breast cancer, HLA class I HC expression was prognostic when stratified for nodal status (Fig. 3) and in a multivariate analysis adjusted for clinical and pathological characteristics (Table 1). In an exploratory analysis within HR+/HER2− breast cancer, HLA class I HC was associated with higher risk of relapse in patients with grade 1–2 tumors, patients with residual disease after neoadjuvant chemotherapy, in patients with non-lobular breast cancer and those without extensive intratumoral lymphocytes (Additional file 1: Figure S1). We also used the percentage of cells positively stained for HLA I to evaluate its association with clinical endpoints without a predefined cut-off (Additional file 3: Table S3).

**Table 1:** Univariate Cox and logistic regression within tumor subtypes and multivariate Cox and logistic regression in patients with HR+/HER2− breast cancer for comparing tumors with high vs. low HLA class I immunohistochemistry

Univariate Cox regression—disease-free survival (GeparTrio)				
Subtype	MHCI	HR	95% CI	*P*
HR+/HER2−	High vs. low	1.590	1.062–2.380	0.024
HR−/HER2−	High vs. low	0.649	0.315–1.336	0.241
HR+/HER2+	High vs. low	2.486	0.956–6.467	0.062
HR−/HER2+	High vs. low	1.015	0.304–3.384	0.981
Multivariate Cox regression—disease-free survival (GeparTrio; HR+/HER2−)				
		HR	95% CI	*P*
Response	pCR vs. RD	0.457	0.225–0.925	0.029
cT stage	cT3–4 vs. cT1–2	2.270	1.522–3.385	< 0.001
cN stage	cN+ vs. cN−	2.026	1.335–3.075	0.001
Therapy	Resp. guided vs. standard	0.945	0.638–1.400	0.777
Grade	G3 vs. G1–2	1.733	1.024–2.932	0.041
Age	Age ≥ 50 vs. < 50	1.252	0.838–1.870	0.272
HLA class I HC	High vs. low	1.701	1.105–2.618	0.016
Univariate logistic regression—pCR (GeparTrio)				
Subtype	MHCI	HR	95% CI	*P*
HR+/HER2−	High vs. low	2.226	1.154–4.585	0.022
HR−/HER2−	High vs. low	0.852	0.332–2.316	0.744
HR+/HER2+	High vs. low	2.022	0.728–6.590	0.202
HR−/HER2+	High vs. low	0.857	0.220–3.385	0.823
Multivariate logistic regression—pCR (GeparTrio; HR+/HER2−)				
		HR	95% CI	*P*
cT stage	cT3–4 vs. cT1–2	0.946	0.447–1.903	0.880
cN stage	cN+ vs. cN-	1.107	0.579–2.135	0.760
Therapy	Resp. guided vs. standard	0.842	0.444–1.578	0.593
Grade	G3 vs. G1–2	2.804	1.348–5.644	0.005
Age	Age ≥ 50 vs. < 50	0.564	0.297–1.055	0.075
HLA class I HC	High vs. low	2.132	1.057–4.603	0.042

### HLA I expression in an independent dataset

For validation, we used data from a clinically annotated, publicly available dataset of 267 hormone HR+/HER2− breast cancer patients with chemo-endocrine treatment [18] that shares characteristics with the GeparTrio cohort (Additional file 3: Table S2). We evaluated the expression of HLA-A, HLA-B, and HLA-C using the continuous data and defined cut points corresponding to the observed frequencies of high vs. low HLA class I expression in the IHC dataset (Table 2, Fig. 3). HLA-A, HLA-B, and HLA-C were strongly correlated (Additional file 2: Figure S2). HLA-A, but not HLA-B or HLA-C, was associated with longer distant recurrence-free survival but not with response to therapy (Table 2, Fig. 3).

**Table 2:** Univariate Cox and logistic regression analyses to predict distant recurrence-free survival using the dichotomized and continuous expression data of HLA-A, HLA-B, and HLA-C, respectively. HLA-A, but not HLA-B and HLA-C, is predictive for patient survival, but not for response to neoadjuvant treatment

Univariate Cox regression—distant recurrence-free survival (MDACC)				
		HR	95% CI	*P*
HLA-A	High vs. low	2.493	1.131–5.494	0.023
HLA-B	High vs. low	2.042	0.956–4.358	0.065
HLA-C	High vs. low	1.477	0.723–3.016	0.284
Univariate logistic regression—pCR (MDACC)				
		HR	95% CI	*P*
HLA-A	High vs. low	1.722	0.699–4.289	0.234
HLA-B	High vs. low	1.373	0.552–3.383	0.487
HLA-C	High vs. low	1.373	0.552–3.383	0.487
Univariate Cox regression—distant recurrence-free survival (MDACC)				
		HR	95% CI	*P*
HLA-A	Continuous	1.576	1.079–2.303	0.019
HLA-B	Continuous	1.364	0.973–1.912	0.072
HLA-C	Continuous	1.359	0.941–1.965	0.102
Univariate logistic regression—pCR (MDACC)				
		HR	95% CI	*P*
HLA-A	Continuous	1.346	0.857–2.103	0.192
HLA-B	Continuous	1.367	0.887–2.132	0.160
HLA-C	Continuous	1.519	0.948–2.466	0.085

### Association of HLA class I expression with immune cell populations

The association of HLA-A expression with previously described immune-cell-specific metagenes [16] was evaluated in the microarray-based dataset to better understand the association with the immunological tumor infiltrate (Additional file 3: Table S4). Within the HR+/HER− subset, positive correlations of the HLA metagene were found with all signatures (Additional file 3: Table S4). The strongest associations were detected for the T cell signatures (*ρ*_P_ = 0.553), myeloid dendritic cells (*ρ*_P_ = 0.488), and cytotoxic lymphocyte signatures (*ρ*_P_ = 0.471).

### Immune cell populations and patient outcome

**Fig. 4:**
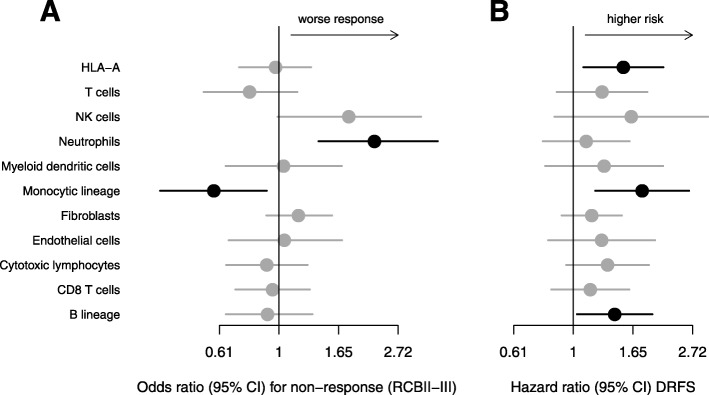
Immune cell populations and patients’ outcome in the HR+/HER2− MDACC cohort: The figure shows the results of univariate logistic regressions (**a**) for the probability of a high residual cancer burden after neoadjuvant therapy (RCBII-III) and the results of univariate Cox regressions (**b**) for distant recurrence-free survival in dependence of immune cell metagene expression. The metagene for neutrophils was associated with lesser response, the metagene representing monocytes with better response. HLA-A and the metagenes for monocytes and the B lineage were associated with higher risk of distant relapse. Statistical significance (*P* < 0.05) is indicated by black points and bars

Univariate Cox and logistic regression were performed to evaluate the predictive and prognostic influence of the different immune cell populations in HR+/HER2− breast cancer. The metagene representing neutrophils was associated with reduced response to neoadjuvant chemotherapy, while the metagene for the monocytic lineage was associated with better response (Fig. 4). The HLA-A metagene, the monocytic lineage, and the B lineage were associated with higher risk of distant relapse.

### Association of HLA class I expression with ESR1 and Aurora kinase A expression

To evaluate subtyping effects of HLA-A expression, we correlated the expression with estrogen receptor 1 (ESR1) and aurora kinase A (AUKRA) as a marker of proliferation [20]. There was no strong association of HLA-A with either gene (*ρ*_P_ = − 0.164 and − 0.122, respectively; Additional file 3: Table S4).

## Discussion

HLA class I HC expression as evaluated by IHC is predictive for better response and worse DFS and OS in HR+/HER2− breast cancer in a large clinical trial cohort. It was positively associated with TILs and independently prognostic for patient survival adjusted for other clinical and pathological factors. It was neither predictive nor prognostic in TNBC or HER2+ disease. We confirmed the prognostic value of HLA-A expression in an independent microarray-based dataset.

HLA class I HC expression was associated with clinical and pathological tumor characteristics indicative of a more aggressive tumor biology such as the presence of lymph node metastases and high histopathological grade. We considered that this might explain the better response, but worse DFS that was associated with high HLA class I HC expression. However, there was no strong association of HLA-A expression with either ESR1 expression or AURKA expression. HLA was prognostic in a multivariate analysis adjusted for clinical and pathological tumor characteristics including nodal status and tumor grade. Thus, the effects could not be explained by these correlations alone.

The main caveat of the study is that we cannot evaluate the role of HLA class I expression for sensitivity to different treatments, as patients received both cytotoxic and endocrine therapy. Also, we cannot provide data on HLA class I in its most relevant context of therapeutic immunomodulation.

Another major limitation of the study is the lack of an independent validation cohort, especially to validate the cut-point used for immunohistochemistry. Even though the GeparTrio and MD Anderson datasets share clinical and pathological features, a different technology is used to detect biomarkers on different biological levels. This approach comes with limitations, both on the technical and clinical levels. An important clinical difference is that in GeparTrio, hormone receptor positivity was defined as ≥ 10% of tumor cells stained for estrogen or progesterone receptor, in the MD Anderson cohort, as any stained tumor cells. As it is known that low hormone receptor-positive cases may resemble triple-negative disease on the molecular level and clinically [21, 22], this might influence the comparability of the cohorts. We have therefore applied an additional filter to exclude cases with low ESR1 expression from the MD Anderson dataset.

With these limitations in mind, we believe it is a strength of our study that we are able to show the prognostic relevance of HLA in different cohorts and on the protein as well as mRNA level, which points out the role of HLA as a relevant parameter for the description of the tumor microenvironment.

HLA class I expression was previously evaluated by immunohistochemistry in 212 breast cancer samples not stratified for subtype [23]. The frequency of strong staining (32.5%) was comparable to our data, but HLA class I was associated with a longer DFS across subtypes. In other cancers, like colorectal [24] and non-small cell lung cancer [25], HLA class I expression was positively associated with survival.

For patients with an excellent response to chemotherapy, we observed a trend towards better survival if HLA class I HC expression was high. However, this subgroup was very small, as most patients with HR+/HER2− breast cancer do not show a complete response. In our recent meta-analysis of TILs in breast cancer [5], TILs were associated with better response to chemotherapy in HR+/HER2− disease, but shorter DFS and OS. The effect was strongest in large tumors and in patients who did not have an excellent response to chemotherapy. Our observations in this study are in line with our current findings, pointing at an interaction between lymphocytes and cytotoxic and endocrine treatment effects.

All of the analyzed immune cell metagenes were positively correlated with HLA-A expression. Only HLA-A, and the monocytic and B cell lineages were statistically significantly associated with higher risk, but as trends and correlations were similar for the different immune cell populations, we found it not possible to pinpoint HLA-A effects to one or more specific immune cell type based on these data.

Patient outcome in HR+/HER2− disease is a function of resistance and sensitivity to neoadjuvant cytotoxic therapy, adjuvant endocrine treatment, and natural biology. Because response to chemotherapy was better in patients with tumors with high HLA class I, we hypothesized that the shorter DFS might be due to an interaction with endocrine treatment. Tumors with a high TIL density might be less responsive to endocrine treatment by unknown mechanisms unrelated to ESR1 expression. Interestingly, immune gene modules can predict poor antiproliferative response to aromatase inhibitors [26, 27]. In a large study on patients receiving endocrine therapy alone, HLA class I was not prognostic, but the presence of FOXP3-positive cells was associated with better survival [28].

## Conclusions

We demonstrate how HLA class I expression can be used to predict worse outcome in HR+/HER2− breast cancer. The results point at a subtype-specific and potentially treatment-specific role of tumor-immunological processes in breast cancer with different implications in triple-negative and hormone receptor-positive disease. Further studies are necessary to understand the underlying mechanisms as a foundation for the potential application of immunotherapy in HR+ breast cancer.

## Supplementary information

**Additional file 1:** **Figure S1.** HLA class I HC immunohistochemistry in the GeparTrio cohort: In this exploratory subgroup analysis, univariate Cox regression models were fit to evaluate the prognostic impact of the dichotomized HLA class I data on progression-free survival (A) and overall survival (B) in HR+/HER2- breast cancer. HLA class I was a negative prognostic marker for disease-free survival across all patients, in those with a lower tumor grade, in tumors without excellent response to chemotherapy, in patients with no special type histopathology and in patients with tumors without extensive lymphocytes. We observed a trend in the opposite direction for patients with high-grade tumors, those with excellent response to chemotherapy and lobular breast cancer. HLA class I was associated with shorter overall survival in patients without excellent response (B).

**Additional file 2:** **Figure S2.** HLA-A, HLA-B and HLA-C expression in the MDACC microarray cohort: A paired scatterplot illustrating the association of the HLA metagenes. The upper right panels show Spearman’s ρ.

**Additional file 3:** **Table S1.** Patient and tumor characteristics of the German Breast Group GeparTrio cohort that was evaluated by immunohistochemistry for HLA class I HC expression compared to frequencies in the overall study population. **Table S2.** Patient and tumor characteristics of the HR+/HER2- subset of the GeparTrio cohort (immunohistochemistry) and the MD Anderson Cancer Center cohort (Affymetrix U133A microarrays). **Table S3.** Univariate Cox and logistic regression within tumor subtypes and multivariate Cox and logistic regression in patients with HR+/HER2- breast cancer using the percentage of tumor cells positively stained for MHC I. **Table S4.** Correlations (Spearman’s ρ) of the immune cell metagenes with HLA-A expression, ESR1 and AURKA expression as a surrogate marker for proliferation.

## Data Availability

Not applicable.
