# Supplementary figures and images for: Human leucocyte antigen class I in hormone receptor-positive, HER2-negative breast cancer: association with response and survival after neoadjuvant chemotherapy
Source: Breast Cancer Res. 2019 Dec 11;21:142. doi: 10.1186/s13058-019-1231-z (PMC6907189; doi:10.1186/s13058-019-1231-z)

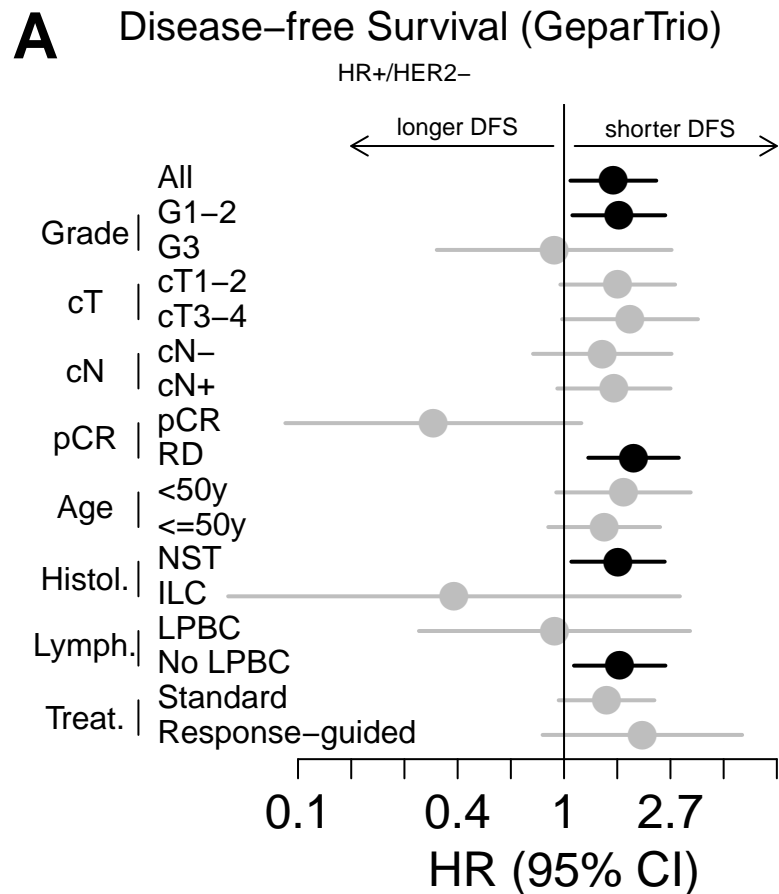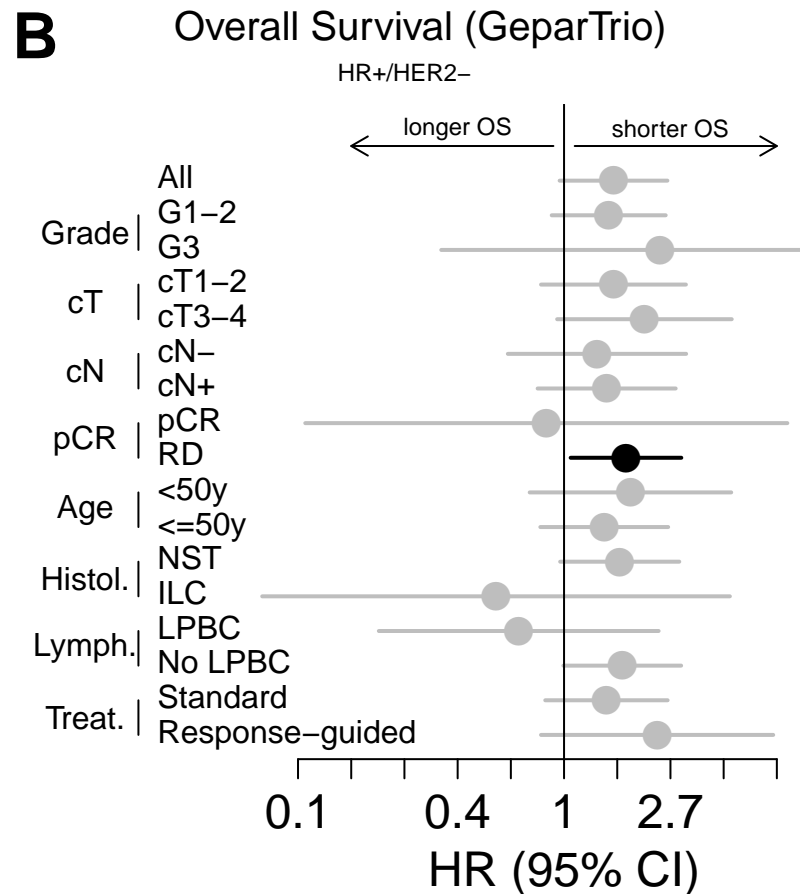

Supplement: Supplementary file 1 — Additional file 1: Figure S1. HLA class I HC immunohistochemistry in the GeparTrio cohort: In this exploratory subgroup analysis, univariate Cox regression models were fit to evaluate the prognostic impact of the dichotomized HLA class I data on progression-free survival (A) and overall survival (B) in HR+/HER2- breast cancer. HLA class I was a negative prognostic marker for disease-free survival across all patients, in those with a lower tumor grade, in tumors without excellent response to chemotherapy, in patients with no special type histopathology and in patients with tumors without extensive lymphocytes. We observed a trend in the opposite direction for patients with high-grade tumors, those with excellent response to chemotherapy and lobular breast cancer. HLA class I was associated with shorter overall survival in patients without excellent response (B). [file 13058_2019_1231_MOESM1_ESM.pdf]

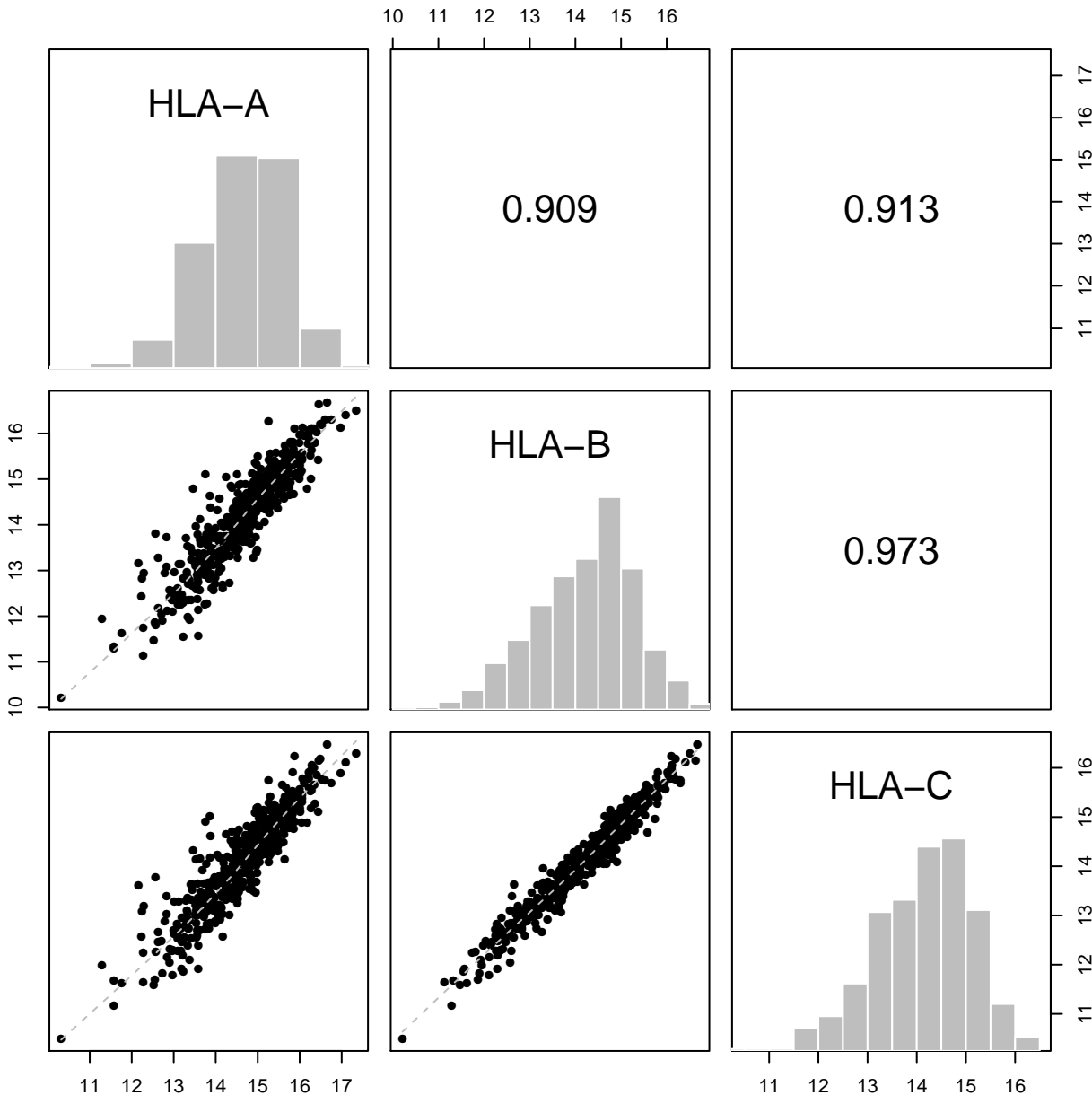

Supplement: Supplementary file 2 — Additional file 2: Figure S2. HLA-A, HLA-B and HLA-C expression in the MDACC microarray cohort: A paired scatterplot illustrating the association of the HLA metagenes. The upper right panels show Spearman’s ρ. [file 13058_2019_1231_MOESM2_ESM.pdf]
