# Supplementary material for: Human leucocyte antigen class I in hormone receptor-positive, HER2-negative breast cancer: association with response and survival after neoadjuvant chemotherapy
Source: Breast Cancer Res. 2019 Dec 11;21:142. doi: 10.1186/s13058-019-1231-z (PMC6907189; doi:10.1186/s13058-019-1231-z)
Supplement: Supplementary file 3 — Additional file 3: Table S1. Patient and tumor characteristics of the German Breast Group GeparTrio cohort that was evaluated by immunohistochemistry for HLA class I HC expression compared to frequencies in the overall study population. Table S2. Patient and tumor characteristics of the HR+/HER2- subset of the GeparTrio cohort (immunohistochemistry) and the MD Anderson Cancer Center cohort (Affymetrix U133A microarrays). Table S3. Univariate Cox and logistic regression within tumor subtypes and multivariate Cox and logistic regression in patients with HR+/HER2- breast cancer using the percentage of tumor cells positively stained for MHC I. Table S4. Correlations (Spearman’s ρ) of the immune cell metagenes with HLA-A expression, ESR1 and AURKA expression as a surrogate marker for proliferation. [file 13058_2019_1231_MOESM3_ESM.docx]

| Table S1 | | | | |
| --- | --- | --- | --- | --- |
| German Breast Group GeparTrio Cohort (IHC) | | | | GeparTrio population |
|  |  | N | % | % |
| HLA class 1 HC | high | 480 | 65.6 | n/a |
|  | low | 252 | 34.4 | n/a |
| Lymphocytes | High | 152 | 20.8 | n/a |
|  | Low | 572 | 78.1 | n/a |
|  | n/a | 8 | 1.1 | n/a |
| Age | >= 50 | 392 | 53.6 | 49.2 |
|  | < 50 | 331 | 45.2 | 50.8 |
|  | n/a | 9 | 1.2 | n/a |
| Tumor type | Ductal/other | 631 | 86.2 | 86.3 |
|  | Lobular | 89 | 12.2 | 13.4 |
|  | n/a | 12 | 1.6 | 0.3 |
| Grade | G1-2 | 565 | 77.2 | 52.8 |
|  | G3 | 166 | 22.7 | 33.6 |
|  | n/a | 1 | 0.1 | 13.6 |
| HR status | HR+ | 544 | 74.3 | 55.9 |
|  | HR- | 159 | 21.7 | 30.2 |
|  | n/a | 29 | 4.0 | 14.0 |
| HER2 status | HER2+ | 145 | 19.8 | 28.9 |
|  | HER2- | 558 | 76.2 | 50.0 |
|  | n/a | 29 | 4.0 | 21.2 |
| cT stage | T1-2 | 480 | 65.6 | 66.1 |
|  | T3-4 | 229 | 31.3 | 31.7 |
|  | n/a | 23 | 3.1 | 2.2 |
| cN stage | cN- | 316 | 43.2 | 43.3 |
|  | cN+ | 383 | 52.3 | 52.8 |
|  | n/a | 33 | 4.5 | 3.9 |
| Treatment | Resp. guided | 340 | 46.6 | 49.1 |
|  | Standard | 383 | 52.3 | 50.9 |
|  | n/a | 9 | 1.2 | n/a |
| Response | pCR | 135 | 18.4 | 16.6 |
|  | RD | 588 | 80.3 | 83.4 |
|  | n/a | 9 | 1.2 | n/a |

**Table S1** Patient and tumor characteristics of the German Breast Group GeparTrio cohort that was evaluated by immunohistochemistry for HLA class I HC expression compared to frequencies in the overall study population.

| Table S2 | | | | | | | | | |
| --- | --- | --- | --- | --- | --- | --- | --- | --- | --- |
| German Breast Group GeparTrio Cohort (IHC) | | | |  | MD Anderson Cancer Center Cohort (microarrays) | | | | |
|  |  | N | % |  |  |  | N | % | |
| HLA class I HC | High | 260 | 59 |  | HLA-A | High | 160 | 60 | |
|  | Low | 177 | 41 |  |  | Low | 107 | 40 | |
| Lymphocytes | High | 61 | 14 |  | Age | >= 50 | 128 | 48 | |
|  | Low | 372 | 85 |  |  | < 50 | 139 | 52 | |
|  | NA | 4 | 1 |  | Grade | G1-2 | 170 | 64 | |
| Age | >= 50 | 242 | 55 |  |  | G3 | 82 | 31 | |
|  | < 50 | 191 | 44 |  |  | NA | 15 | 5 | |
|  | NA | 4 | 1 |  | cT stage | T1-2 | 154 | 58 | |
| Tumor type | Ductal/other | 359 | 82 |  |  | T3-4 | 113 | 42 | |
|  | Lobular | 72 | 16 |  | cN stage | cN- | 93 | 35 | |
|  | n/a | 6 | 1 |  |  | cN+ | 174 | 65 | |
| Grade | G1-2 | 375 | 86 |  | Response | pCR | 21 | 8 | |
|  | G3 | 62 | 14 |  |  | RD | 236 | 88 | |
|  | NA | 0 | 0 |  |  | NA | 10 | 4 | |
| cT stage | T1-2 | 303 | 69 |  |  |  |  |  |  |
|  | T3-4 | 122 | 28 |  |  |  |  |  |  |
|  | NA | 12 | 3 |  |  |  |  |  |  |
| cN stage | cN- | 196 | 45 |  |  |  |  |  |  |
|  | cN+ | 221 | 51 |  |  |  |  |  |  |
|  | NA | 20 | 5 |  |  |  |  |  |  |
| Treatment | resp. guided | 202 | 46 |  |  |  |  |  |  |
|  | standard | 231 | 53 |  |  |  |  |  |  |
|  | NA | 4 | 1 |  |  |  |  |  |  |
| Response | pCR | 48 | 11 |  |  |  |  |  |  |
|  | RD | 385 | 88 |  |  |  |  |  |  |
|  | NA | 4 | 1 |  |  |  |  |  |  |

**Table S2** Patient and tumor characteristics of the HR+/HER2- subset of the GeparTrio cohort (immunohistochemistry) and the MD Anderson Cancer Center cohort (Affymetrix U133A microarrays).

| Table S3 | | | | |
| --- | --- | --- | --- | --- |
| Univariate Cox regression – Disease-free survival (GeparTrio) | | | | |
| Subtype | MHCI | HR | 95 % CI | p |
| HR+/HER2- | % | 1.006 | 0.999 - 1.013 | 0.057 |
| HR-/HER2- | % | 0.997 | 0.986 - 1.008 | 0.630 |
| HR+/HER2+ | % | 1.014 | 1.000 - 1.029 | 0.051 |
| HR-/HER2+ | % | 1.002 | 0.980 - 1.025 | 0.855 |

| Multivariate Cox regression – Disease-free survival (GeparTrio; HR+/HER2-) | | | | |
| --- | --- | --- | --- | --- |
|  |  | HR | 95 % CI | p |
| Response | pCR vs. RD | 0.476 | 0.235 - 0.964 | 0.039 |
| cT stage | cT3-4 vs. cT1-2 | 2.247 | 1.501 - 3.351 | 0.000 |
| cN stage | cN+ vs. cN- | 2.039 | 1.342 - 3.098 | 0.001 |
| Therapy | Resp. guided vs. standard | 0.936 | 0.631 - 1.386 | 0.741 |
| Grade | G3 vs. G1-2 | 1.704 | 0.999 - 2.909 | 0.051 |
| Age | Age >=50 vs. < 50 | 1.271 | 0.851 - 1.898 | 0.242 |
| HLA class I HC | % | 1.007 | 1.000 - 1.014 | 0.063 |

| Univariate Logistic regression – pCR (GeparTrio) | | | | |
| --- | --- | --- | --- | --- |
| Subtype | MHCI | HR | 95 % CI | p |
| HR+/HER2- | % | 1.014 | 1.003 - 1.025 | 0.016 |
| HR-/HER2- | % | 0.994 | 0.980 - 1.008 | 0.424 |
| HR+/HER2+ | % | 1.008 | 0.992 - 1.026 | 0.338 |
| HR-/HER2+ | % | 0.989 | 0.964 - 1.013 | 0.377 |

| Multivariate Logistic regression – pCR (GeparTrio; HR+/HER2-) | | | | |
| --- | --- | --- | --- | --- |
|  |  | HR | 95 % CI | p |
| cT stage | cT3-4 vs. cT1-2 | 0.923 | 0.437 - 1.853 | 0.826 |
| cN stage | cN+ vs. cN- | 1.084 | 0.566 - 2.093 | 0.809 |
| Therapy | Resp. guided vs. standard | 0.811 | 0.426 - 1.520 | 0.516 |
| Grade | G3 vs. G1-2 | 2.740 | 1.311 - 5.542 | 0.006 |
| Age | Age >=50 vs. < 50 | 0.569 | 0.300 - 1.065 | 0.080 |
| HLA class I HC | % | 1.013 | 1.001 - 1.025 | 0.036 |

**Table S3** Univariate Cox and logistic regression within tumor subtypes and multivariate Cox and logistic regression in patients with HR+/HER2- breast cancer using the percentage of tumor cells positively stained for MHC I

| Table S4 | | | |
| --- | --- | --- | --- |
| Table 5. Correlation of HLA-A with immune cell metagenes | | | |
| Cell population | HLA-A | ESR1 | AURKA |
| B lineage | 0.433 | -0.129 | -0.028 |
| CD8 T cells | 0.361 | -0.003 | -0.050 |
| Cytotoxic lymphocytes | 0.471 | -0.189 | 0.037 |
| Endothelial cells | 0.211 | -0.127 | -0.204 |
| Fibroblasts | 0.204 | -0.072 | -0.245 |
| Monocytic lineage | 0.440 | -0.434 | 0.029 |
| Myeloid dendritic cells | 0.488 | -0.185 | -0.046 |
| Neutrophils | 0.251 | 0.030 | -0.157 |
| NK cells | 0.295 | -0.044 | -0.178 |
| T cells | 0.553 | -0.176 | -0.038 |
| HLA-A | 1 | -0.164 | -0.122 |

**Table S4** Correlations (Spearman’s ρ) of the immune cell metagenes with HLA-A expression, ESR1 and AURKA expression as a surrogate marker for proliferation.
